# Supplementary material for: Characterization of Meat Metabolites and Lipids in Shanghai Local Pig Breeds Revealed by LC–MS-Based Method
Source: Foods. 2024 Jul 24;13(15):2327. doi: 10.3390/foods13152327 (PMC11312277; doi:10.3390/foods13152327)
Supplement: Supplementary file 1 [file foods-13-02327-s001.zip › Supplementary Material S1.pdf]

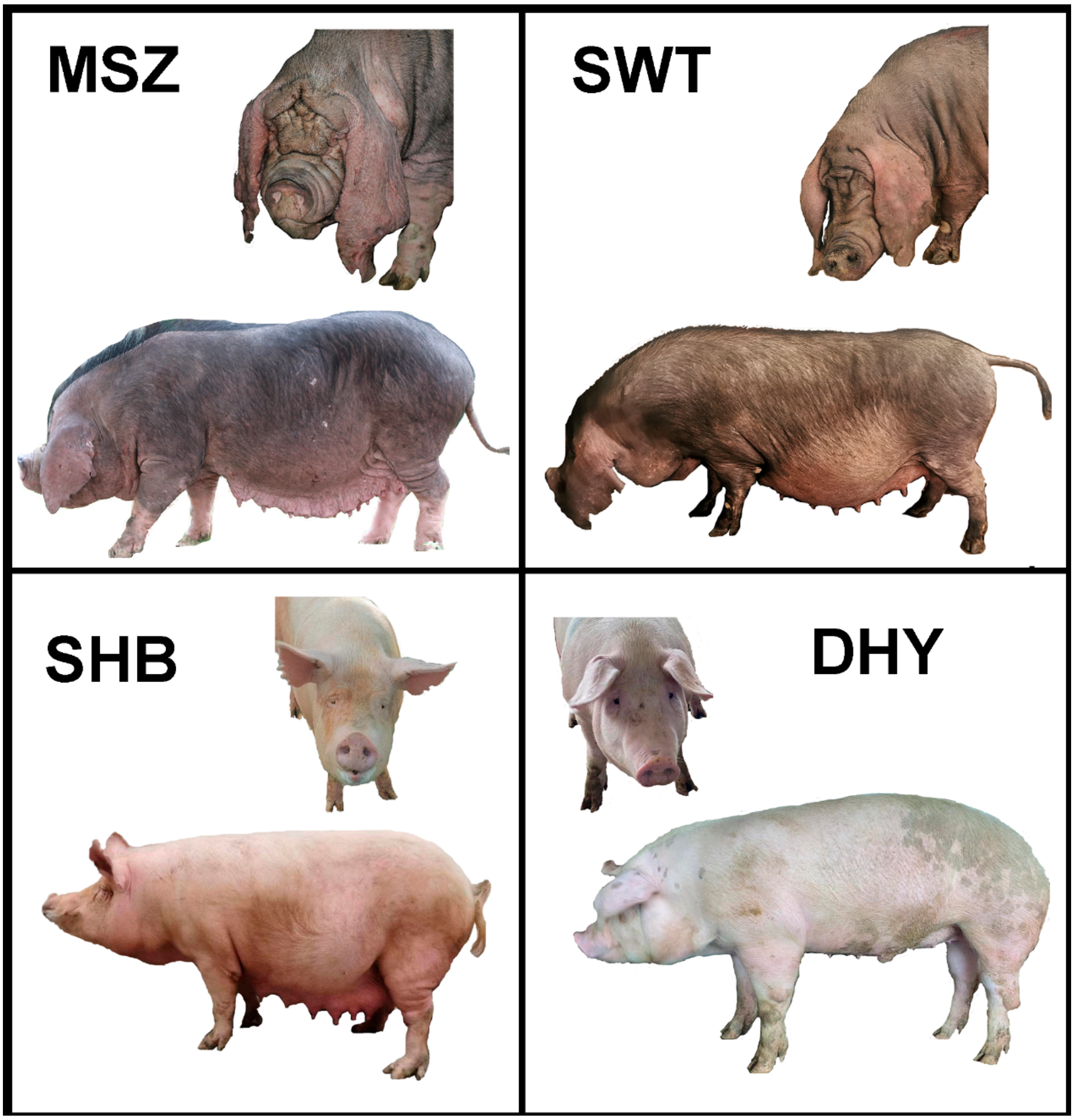

Figure S1. Appearance pictures of the four pig breeds in this study. Main ingredients of feed formulations for the pigs: Crude protein 18.0%, Leucine 0.63%, Energy/MJ·kg 13.9%, Methionine 0.29%, Calcium 0.65%, Threonine 0.56%, Total phosphorus 0.49%, Glutamic acid 2.91%, Lysine 0.85%, and Crude fiber 4%.
